# Mathematical Modeling and COVID-19 Forecast in Texas, USA: A Prediction Model Analysis and the Probability of Disease Outbreak

**DOI:** 10.1017/dmp.2021.151

**Published:** 2021-05-19

**Authors:** Md Nazmul Hassan, Md. Shahriar Mahmud, Kaniz Fatema Nipa, Md. Kamrujjaman

**Affiliations:** 1 Department of Mathematics and Statistics, Texas Tech University, Lubbock, Texas, USA; 2 Department of Sciences and Mathematics, Schreiner University, Kerrville, Texas, USA; 3 Department of Computer Science and Engineering, State University of Bangladesh, Dhaka, Bangladesh; 4 Department of Mathematics, University of Dhaka, Dhaka, Bangladesh; 5 Department of Mathematics and Statistics, University of Calgary, Calgary, AB, Canada

**Keywords:** SEIR model, COVID-19, Texas, Continuous-Time Markov Chain (CTMC), parameters

## Abstract

**Background:**

Response to the unprecedented coronavirus disease 2019 (COVID-19) outbreak needs to be augmented in Texas, United States, where the first 5 cases were reported on March 6, 2020, and were rapidly followed by an exponential rise within the next few weeks. This study aimed to determine the ongoing trend and upcoming infection status of COVID-19 in county levels of Texas.

**Methods:**

Data were extracted from the following sources: published literature, surveillance, unpublished reports, and websites of Texas Department of State Health Services (DSHS), Natality report of Texas, and WHO Coronavirus Disease (COVID-19) Dashboard. The 4-compartment Susceptible-Exposed-Infectious-Removal (SEIR) mathematical model was used to estimate the current trend and future prediction of basic reproduction number and infection cases in Texas. Because the basic reproduction number is not sufficient to predict the outbreak, we applied the Continuous-Time Markov Chain (CTMC) model to calculate the probability of the COVID-19 outbreak.

**Results:**

The estimated mean basic reproduction number of COVID-19 in Texas is predicted to be 2.65 by January 31, 2021. Our model indicated that the third wave might occur at the beginning of May 2021, which will peak at the end of June 2021. This prediction may come true if the current spreading situation/level persists, i.e., no clinically effective vaccine is available, or this vaccination program fails for some reason in this area.

**Conclusion:**

Our analysis indicates an alarming ongoing and upcoming infection rate of COVID-19 at county levels in Texas, thereby emphasizing the promotion of more coordinated and disciplined actions by policy-makers and the population to contain its devastating impact.

Coronavirus disease 2019 (COVID-19) is a serious global health threat. On March 11, 2020, the World Health Organization (WHO) declared COVID-19 a pandemic, as it spreads worldwide rapidly following the logistic growth pattern.^1^ More than 103 million cases were reported worldwide by January 31, 2021, where the United States of America (USA) has over 26.5 million cases alone. The virus that causes the COVID-19 disease is known as severe acute respiratory syndrome coronavirus 2 (SARS-CoV-2). SARS-CoV-2 is spreading very quickly in the human population. The main route of spreading this virus is close person-to-person contact, and the spread is sustainable, as it goes from person-to-person without stopping. The ongoing COVID-19 pandemic is spreading more efficiently than influenza, but not as efficiently as highly contagious measles.^2^ Another complicating factor is that asymptomatic patients are still able to spread the virus. According to the Centers for Disease Control and Prevention (CDC), common symptoms include but are not limited to fever or chills, cough, shortness of breath or difficulty breathing, sore throat, and many more.

As infected populations and the death tolls continue to rise rapidly, governments worldwide are trying to control the pandemic by reducing people’s close contact, such as shutting down public places, schools, colleges, universities, restaurants, playgrounds, and the list continues. Due to the lack of proper viral medicine or vaccine, travel bans from highly infected areas, social distancing, lock-down policy, isolation of an infected person, self-quarantine of exposed individual, mandatory use of face masks or face coverings, and strictly following all social-conscious and prevention strategies^3^ have been widely used strategies to contain the virus.

Infectious disease modeling is one of the most critical parts of understanding the current pandemic’s ongoing scenario. Mathematical modeling techniques, particularly the compartmental modeling technique, can help us estimate crucial parameters, such as disease transmission dynamics, number of infected people, number of hospitalized people, and number of recovered/dead individuals. Consequently, it helps to forecast outbreak timelines and the overall dynamics of the disease. The knowledge gained from mathematical modeling helps determine strategies to mitigate the outbreak and determine whether the interventions taken are useful or not.

The modeling community took the challenge to face the COVID-19 pandemic together. It started modeling to understand the dynamics of COVID-19 and help determine the intervention to slow the spread of the virus. Most of the models related to COVID-19 use population-based, Susceptible-Infected-Removal (SIR) models with differential equations or stochastic differential equations^4–7^ or susceptible-Exposed-Infectious-Removal (SEIR) and extended SEIR compartmental models.^9–18^ Some agent-based models developed related to COVID-19 use structured networks to connect individuals and model infection exchanges stochastically.^19–23^

Mathematical models often made redundant use of parameters and equations. Our primary focus is to keep the model as easy and straightforward as possible, so it will be understandable to the nonmath major population for a broader perspective. In this study, we develop a deterministic 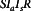 (modified SEIR) model to deepen our understanding of COVID-19’s dynamic. Theoretically, we analyze the model to show the existence and positivity invariance of the system’s solutions and determine the Disease-Free Equilibrium (DFE) and Endemic Equilibrium (EE). We calculate the basic reproduction number, 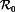 by using the next-generation matrix approach. We also analyze the local stability of the DFE and EE fixed points. Finally, we estimate the probability of an outbreak using the Continuous-time Markov Chain (CTMC) model for better prediction to control the disease outbreak.

We parameterize our model for Texas, United States, by using the data from March 6, 2020, to January 31, 2021, available at https://www.dshs.texas.gov/coronavirus/.^24^ Using numerical simulations and data analyses, we predict the daily confirmed case and the cumulative case of Texas and compare the model prediction with the existing data.^25^ We provide a sensitivity and elasticity index analysis of 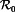 to understand better the most fluctuating (sensitive) parameter of the model. Furthermore, in this study, we developed the CTMC model. We estimate the probability of a disease outbreak depending on the most critical parameter of the model to determine better control measures of the diseases. The main objectives of the study are: (a) We will work with real-life available discrete data of Texas to understand the cases and project the control of the infection; and (b) we expect that the prediction of controlling measure of COVID-19 will be able to validate the dynamics of the 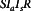 and CTMC models to obtain more accurate results.

We organized this article as follows: Mathematical Model discussed elaborately with positivity and boundedness of solution in the Mathematical Model and Existence of Solutions section. The fixed points, auxiliary results are described in the Determination of Fixed Points section. The local stability analysis, parameter estimation, and sensitivity analysis described in the Stability Analysis, Parameter Estimation, and Sensitivity section. The data analysis compared with the model solution with further prediction to control the epidemic, as a case study in Texas accomplished in the Numerical Simulation and Results section. The probability of disease outbreak with CTMC analysis presented in the Probability of a Disease Outbreak section. Finally, the Concluding Remarks section outlines the summary and concluding remarks of the results.

## Mathematical Model and Existence of Solutions

The classical SIR model predicts the dynamics of infectious disease. We started with the following compartmental SIR model proposed in Murray^25^:(2.1)
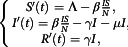
for 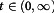 with initial conditions(2.2)
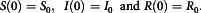
and for total population, 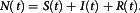


Here 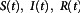 are the number of individuals in the susceptible, infected and removed compartments, respectively at time 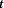 with a day unit. The parameter 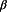 denotes the infection rate/disease transmission rate, and 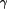 and 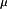 are the removal and disease induced mortality rate, respectively. The solution and detailed analysis of Equation (2.1) are available in Murray.^25^

Compared with the SIR epidemic model, the next updated and advanced model is SEIR, which is biologically more feasible in many pandemics and infectious diseases. In this study, we consider the following 4 compartments 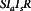 mathematical model; a modified version of the typical SEIR model:(2.3)
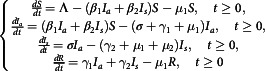
with initial conditions(2.4)
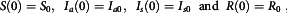
and(2.5)
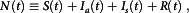



**Figure 1.**
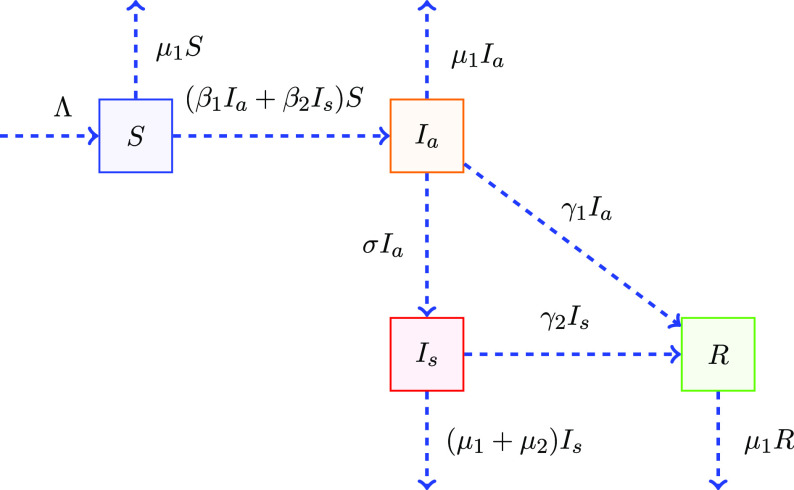
Compartmental diagram for model.

Here, 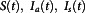 and 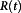 are the number of individuals in the susceptible, asymptomatically infected, symptomatically infected (for simplicity, we will call the symptomatically infected population as an infected population), and removed compartments, respectively, at time 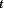 per day unit. 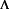 is the recruitment number in the susceptible compartment. Natural and disease induced deaths are denoted by 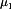 and 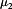, respectively. 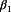 and 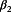 are the diseases transmission rates of susceptible individuals with asymptomatically infected and infected ones, respectively, which may cause the transmission of the infection, 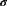 is the transition rate from asymptomatically infected to infected compartment and 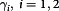 are the recovery rates from asymptomatically infected and infected to removal compartment, respectively. After transmission, a susceptible individual initially becomes asymptomatically infected. As the disease progresses, asymptomatically infected individuals may develop symptoms, and transition from 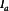 to 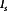 at rate 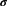 or they may never develop symptoms and recover at rate 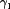. Because the model monitors dynamics of population, it follows that all its dependent variables and parameters, for example, 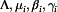 and 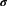 must be non-negative along with 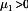 as in the model Equations (2.3)-(2.5). The definition of all parameters are elaborated in Table 1 and the flow diagram of the main model Equation (2.3) is shown in Figure 1.

**Table 1.** Model parameters and their descriptions

Notation	Definition	Notation	Definition
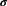	Transition rate from 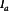 to 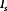 class	λ	Recruitment rate in *S* class
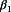	Transmission rate from contact with 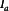	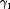	Recovery rate of 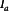 class
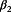	Transmission rate from contact with 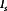	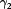	Recovery rate of 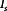 class
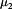	Disease induced death rate	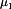	Natural death rate

Remark 1 *In this study, we consider the modified SEIR model for SARS-CoV-2 dynamics to cover both infectious and exposed-asymptomatic individuals compared to the classical SIR and SEIR compartment models. The model has a combined compartment for exposed and asymptomatic classes, which reduces the number of parameters and eventually reduces the model’s complexity.*

The following result ensures the existence and positivity of solutions of Equation (2.3).

Theorem 1 *The closed region* 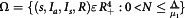 *is positively invariant set for the system in* Equation (2.3).

The proof of theorem 1 can be found in the Appendix.

## Determination of Fixed Points

To find the equilibrium points 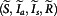 of the system Equation (2.3), we set the derivatives equal to zero. So, at equilibrium states, we get(3.1)
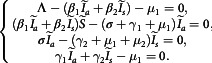



### DFE Point

For the DFE, we replace the variables as
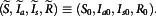



This gives,
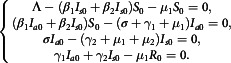



Therefore, the DFE point can easily be found as(3.2)
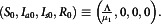



### EE Point

For the EE, we replace the variables as 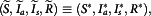 where, 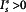. And we have the following system(3.3)
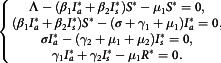



Then the third equation of the system Equation (3.3) gives,(3.4)
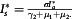



Similarly, the fourth equation yields(3.5)
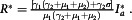



Next, the first equation of Equation (3.3) yields(3.6)
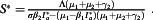



Finally, the second equation of Equation (3.3) gives(3.7)
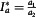
where,
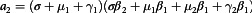


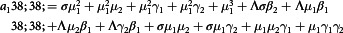



Hence the endemic steady state is completely depending on 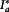.

## Basic Reproduction Number Using Next-Generation Matrix

In this section, we calculated the basic reproduction number, which is a crucial threshold in analyzing infectious disease modeling. It regulates whether the disease will die out or persist in the population.^26,27^ The basic reproduction number, denoted 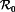, “the expected number of secondary cases produced, in a completely susceptible population, by a typical infective individual”.^28^ If 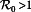, the DFE is unstable, which means 1 primary infection can produce more than 1 secondary infection and epidemic breaks out. If 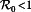, the DFE is locally asymptotically stable, the disease cannot persist in the population, and the situation is sustainable.

In this manuscript, we have used the next generation matrix method^8^ to find basic reproduction number of the system Equation (2.3). We obtain 2 following matrix from the system Equation (2.3), which are *F* and *V*, they are given below
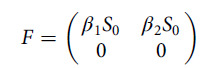
and
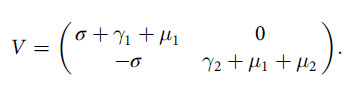



Therefore, the 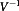 matrix is
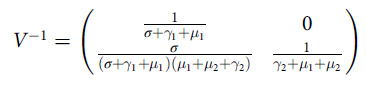



Thus, the next-generation matrix 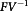 is
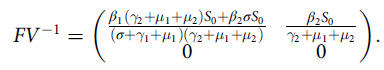



Hence, the basic reproduction number 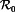 is
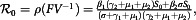

(3.8)
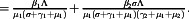



We also have determined the Jacobian matrix of the system Equation (2.3) at any equilibrium point 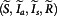 which will be used for further analysis. which will be used for further analysis. The Jacobian matrix of the system Equation (2.3) is given by(3.9)
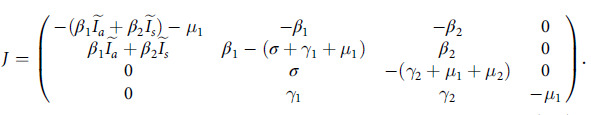



### Stability Analysis, Parameter Estimation, and Sensitivity

Initiallly, we have studied the stability analysis at the DFE point and the EE point and the statement of the results are as follows.

Theorem 2 *The DFE* 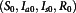 *of* Equation (2.3) *is locally stable if* 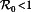 *and unstable if* 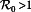.

The proof of theorem 2 can be found in the Appendix.

Theorem 3 *The EE* 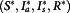 *of the system* Equation (2.3) *is locally stable if* 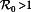.

The proof of theorem 3 is available in the Appendix.

### Parameter Estimation

We used the Texas data from March 6, 2020, to January 31, 2021 (available at https://www.dshs.texas.gov/coronavirus/) ^24^ to estimate the parametric values. The transmission rates 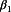 and 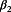 are estimated as piece-wise function values according to the mutation behavior of COVID-19 virus using the source data in https://www.dshs.texas.gov/coronavirus/ ^24^ and https://www.usapopulation.org/texas-population, ^29^ given as a possible interval in Table 2, initially making an assumption on the asymptomatically infected class population. The recruitment rate in *S* class, daily natural deaths, and the total population of Texas are collected from https://www.usapopulation.org/texas-population.^29^ We used the average incubation period, - to -d interval, to estimate the disease transition from 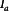 to 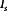 class, 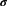. The recovery rates 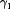 and 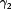 from 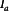 and 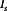 compartments are calculated using the 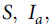 and 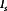 class source data (available in https://www.dshs.texas.gov/coronavirus/ ^24^ and https://www.usapopulation.org/texas-population ^29^), with total recovery and total cases. The disease induced death rate is estimated following the formula of WHO.^30^

**Table 2.** Model parameters values and sensitivity index

Notation	Definition	Value	Source	Numerical Elasticity
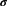	Transition rate from 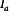 to 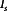	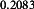 day 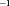	Estimated	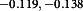
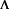	Recruitment rate in *S* class	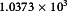 day 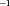	[30]	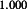
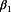	Transmission rate from contact with 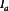	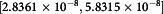 day 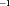	Estimated	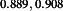
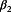	Transmission rate from contact with 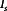	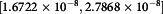 day 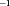	Estimated	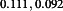
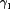	Recovery rate of 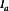	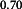 day 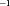	[25]	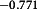
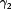	Recovery rate of 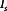	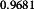 day 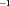	[25]	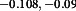
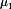	Natural death rate	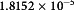 day 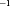	[30]	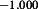
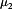	Disease induced death rate	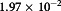 day 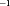	[25]	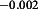
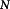	Total Population in Texas	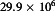	[30]	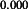

### *Sensitivity and Elasticity Index of* 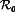


Because 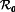 provides qualitative information of an infectious disease modeling, the sensitivity and elasticity of 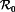 can play an important role in determining a disease’s control strategy. The sensitivity index of 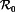 with respect to any parameter, *u* is defined by 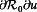. The elasticity index, also known as the normalized sensitivity index of 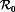, measures the relative change of 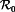 with respect to a parameter. The elasticity index of 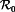 with respect to any parameter, *u* is defined by
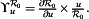




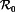
 increases with the positive sign of the elasticity index of the parameter and decreases with the negative sign. The magnitude of the elasticity index tells us the importance of the parameter. These measures used to determine the control of the parameters of an epidemic model. More examples can be found in Van den Driessche.^31^ The numerical elasticity index of 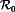 for the baseline parameter values is provided in Table 2.

We observe that the models’ most critical parameters are the 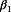 and 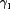 with the average value of 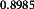 and 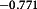 (see sensitivity index in Table 2), respectively. An effective way to control the outbreak could be controlling the parameters 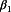 and 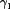. As 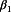 represents the transmission rate from *S* to 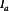 class with the contact of 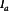, the reduction of contact between the asymptomatic individual to the general population can play an important role. One of the proven measures is to use a face mask uniformly for all populations,^32^ significantly reducing the contact between the classes and reducing disease transmission. If doctors can find some effective viral medicine to improve the recovery rate, it could be an effective way to control the disease.

## Numerical Simulation and Results

To reach the first 100,000 cases, Texas took 105 d, the second hundred thousand cases confirmed withing the next 18 d, but third, fourth, and fifth hundred thousand cases took only 11, 12, and 13 d, respectively. It is noticeable that in the month of January 2021, Texas reported over a half million confirmed cases of COVID-19 and approximately 4000 deaths.^24^

The exact data also show that the second wave came almost after day 90 of the first infection wave, and during the second wave, the peak of daily case confirmation reached up to 14,916 on day 134 of pandemic. Again, the third wave had hit the locals approximately at day 250, with a peak of 28,020 on day 299. We assumed 2 major and highly effective mutations of the malign virus all-around Texas, and so imposed 3 impulsed values for the transmission rates 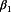 and 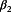.

### Current Epidemic Situation of COVID-19 at Texas State

**Figure 2.**
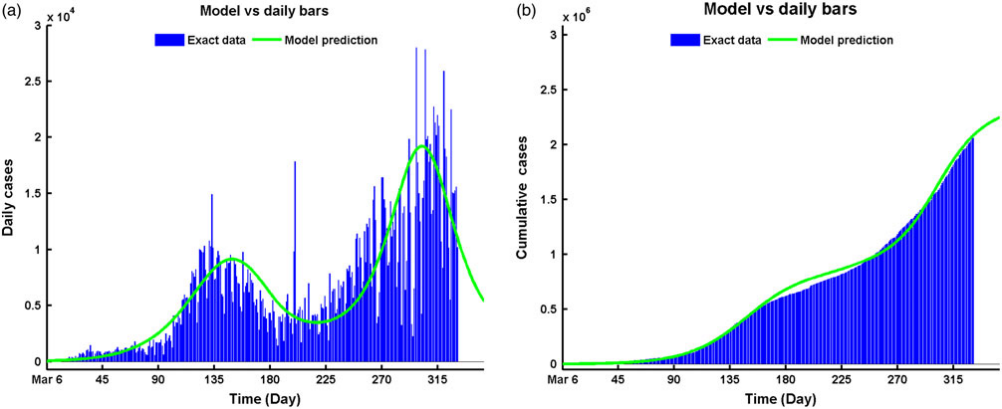
Comparative solutions between data and model prediction of Equation (2.3) for (a) daily cases vs model, and (b) cumulative vs model.

In this subsection, we provide the numerical results of our proposed model and compare the results with the data of Texas.^24^ Figure 2 presents the current epidemic situation of COVID-19 in Texas. The highest number of daily new confirmed cases of COVID-19 reported during December 29, 2020, and January 26, 2021 (Figure 2a). Our model predicted the same number of daily new cases compared with the daily new cases reported by https://www.dshs.texas.gov/coronavirus/.^24^ As of January 31, 2021, Texas has a total of 2,059,143 cases, and our model predicted 2,081,753 total cases for the same date (Figure 2b).

### Projecting the Epidemic Situation of COVID-19

**Figure 3.**
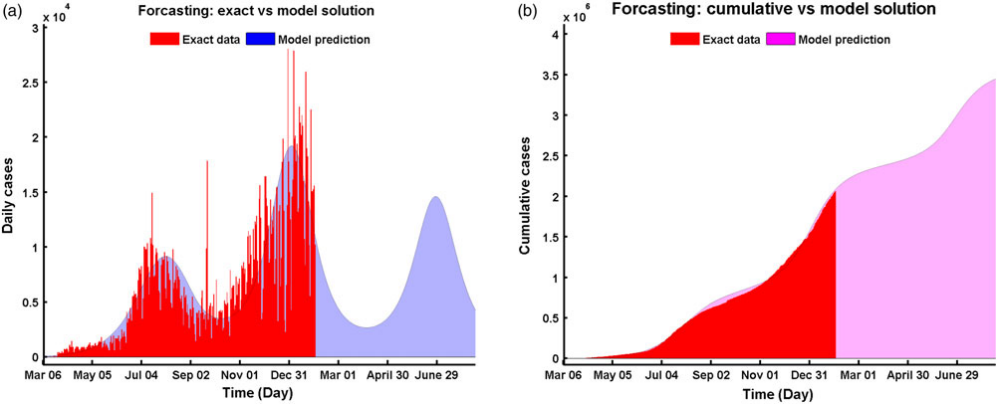
Comparative solutions between data and model prediction of Equation (2.3) for (a) daily cases vs model solution, and (b) cumulative data vs. model solution.

The proposed model predicts another wave (third wave) to happen at the beginning of May 2021, as depicted in Figure 3a, which will have its peak at the end of June 2021. This prediction may come true if the current situation persists, ie, no clinically effective vaccine is available. But the hope is, vaccination has been started in Texas at the beginning of this Spring 2021.^32^ Moreover, if this vaccination program fails, there will be 3,439,804 cases of infection after July 2021 (Figure 3b).

**Figure 4.**
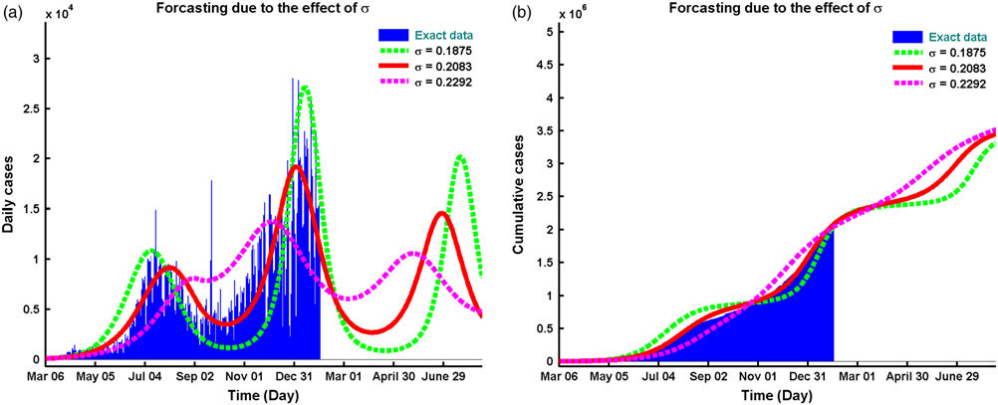
Forecasting due to the effect of using model solution of Equation (2.3) for (a) daily and (b) cumulative cases.

When 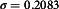, we observe the predicted peak of daily new cases is 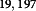 on January 03, 2021, day 303 of the pandemic. If we increase 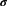 by 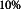 to 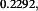 then the peak for the daily new cases moved to December 05, 2020, as 13,757 which also makes the third wave happen earlier. When we decrease 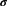 by 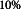 to 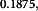 then the peak for the daily new cases moved to January 14, 2021, as 27,149. This 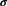 reduction also delays the next wave as depicted in Figure 4a. We see similar dynamics for the cumulative cases (Figure 4b). So 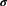 plays a vital role in the dynamics of the epidemics.

It forecasts that on the baseline value 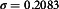 by the end of July 2021, Texas may have 3,439,804 million infected cases of COVID-19. If we increase 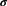 by 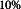 to 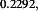 then the total infected cases increases from 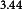 to approximately 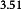 million (3,509,384). If we decrease the value of 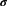 by 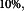 then it reduces to 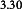 million (3,304,451) infections (see Figure 4b).

**Figure 5.**
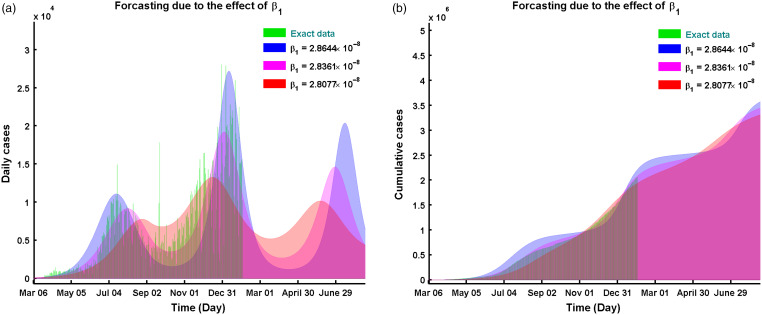
Forecasting due to the only 1% changing effect of using model solution of Equation (2.3) for (a) daily cases, and(b) cumulative cases.

By simulating the transmission rate from the susceptible individuals to the asymptomatic individuals, 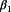, we notice a little bit of change of 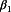 plays a vital role in the forecast of the entire epidemic. For example, an increase in 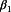 by only 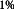 delays the next third wave for almost 20 d, but costs more lives than the base value of 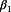. The base line shows 122,492 more cases than the base line solution outcomes. On the other hand, deduction of 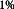 in 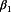 from the base value predicts predicts 135,103 fewer cases in total than when 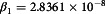 (see Figure 5b). Again, it is clear that these changes in 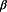 cannot resist the third wave at any cost; the comparison is proved in Figure 5a. Hence, because at the time of this writing, the COVID-19 vaccine has yet to distributed to a significant number of people, the most effective way to prevent the virus is to maintain social distancing and to use a face mask (recommended by the Eikenberry et al.^33^).

**Figure 6.**
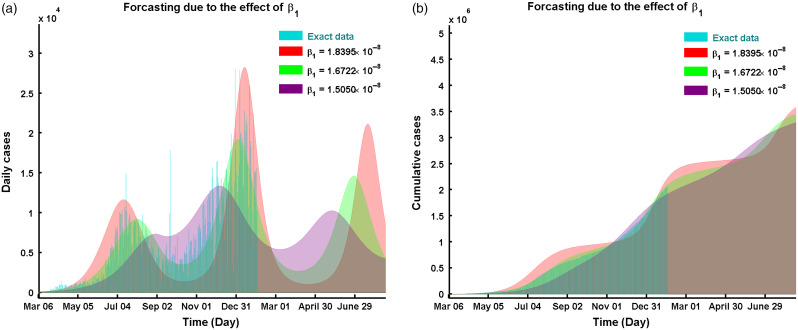
Forecasting due to the 10% changing effect of using model solution of Equation (2.3) for (a) daily cases, and (b) cumulative cases.

Similarly, decreasing 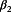 by 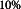 will shorten the interval between the concurrent waves. However, the total number of cases does not change significantly because of the difference between the wave heads and bottom-lands. It shows fewer cases (159,450) than the base model fitting simulation (see Figure 6b). Again, a 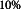 raise at 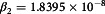 makes the waves a little bit out-lying than the other values in Figure 6a and makes the peaks sharper. It predicts 127,946 more cases in total until July 2021 (Figure 6b).

From above, it is now clear that, to get a similar type of epidemic change, we needed to change 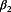 almost 10 times compared to 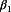. The contact between *S* to 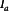 class is random instead of *S* to 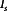 class.

**Figure 7.**
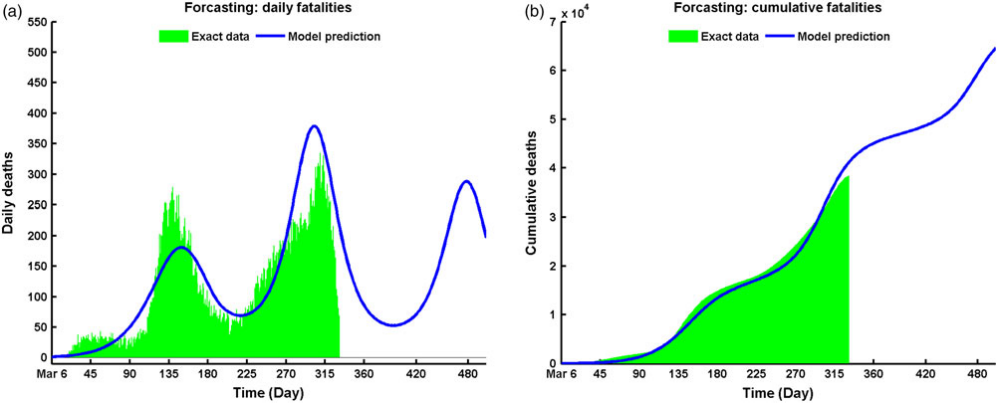
Numerical solutions and data fitting for (a) daily deaths, and (b) cumulative deaths.

Figure 7 presents the daily and cumulative death rate in Texas with the best data fitting results. On the peak day, the model solution predicted the maximum daily death count was people on the last week of December 2020 (see Figure 7a). The total number of dead was approximately 41,000 by February 2021 (Figure 7b), and predicted to be by July 2021 if the spread is not under control already.

### Probability of a Disease Outbreak

#### CTMC Model

To derive a continuous-time Markov chain model assumed 4 random variables for 4 states 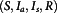 of the deterministic 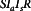 mathematical epidemic model. The variables are discrete-valued and time is continuous, 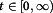,
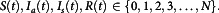



For the simplification of the notations, we use the same notations as we used in the mathematical model to define the infinitesimal transition probabilities, given in Table 3. Let 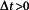 be sufficiently small so that at most one event occurs during the 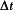 time interval. Let 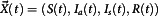 and 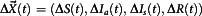, where 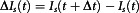, etc.

**Table 3.** Infinitesimal transition probabilities for the 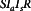 mathematical model

Event *i*	Description	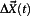	Probabilities 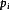
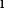	Natural birth	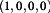	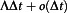
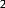	Transmission from *S* to 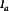	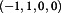	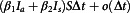
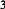	Natural death	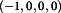	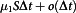
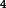	Asymptomatic exposed infection	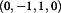	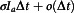
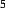	Asymptomatic natural death	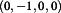	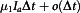
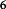	Asymptomatic recovery	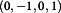	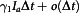
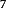	Recovery	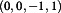	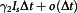
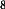	Natural and disease related death	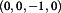	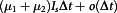
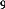	Recovered death	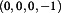	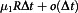
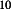	No changes	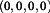	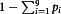

For example, event 2 is a new infection and the probability of a new infection in time 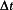 is,
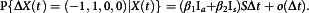



To estimate the probability of a disease outbreak from the CTMC model, the effect of transmission rates from *S* to 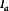 and from *S* to 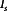, which is 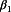 and 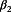 are considered. To approximate the probability of an outbreak, 5000 sample paths are simulated for 1 initial infected individuals, and the simulation will stop if either 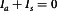 or 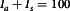 is reached. If the total asymptomatically infected and infected population reaches 100, it is counted as an outbreak. When it reaches zero, it is assumed that there is a probability of extinction. The calculated probability of extinction is a proportion of 5000 sample paths as 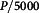. Then the probability of an outbreak will be 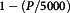. Parameter values are shown in Table 2. The value of 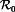 is computed from the expression of Equation (3.8).

Table 4 records the basic reproduction numbers and the probability of an outbreak for a set of values of 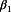 and 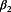. The outbreak value increases when the transmission rate from the susceptible to asymptomatically infected class and from the susceptible to infected class increases. It is obvious that, when the transmission from susceptible to infected populations increase, the risk of outbreak will increase. But for the transmission from the susceptible class to the asymptomatically infected class, there is a threshold value for 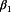, not for 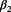. Here, 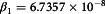 serves as a threshold parameter value for the disease outbreak of the ordinary differential equation system Equation (2.3) when all other parameter are fixed. Also, the probability of an outbreak depends on the initial number of infected populations.^34^ For our model parameter values, the basic reproduction number is 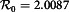 and the probability of an outbreak is 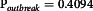.

**Table 4.** Basic reproduction number and the probability of an outbreak are computed from the CTMC model for different value of 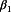 and 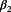. Initial number of infected and asymptomatically infected populations are 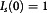 and 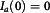

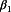	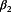	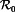	
Only 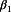 varies			
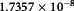	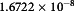	1.3875	0.2758
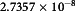	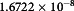	2.0087	0.4094
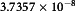	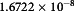	2.6297	0.4704
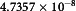	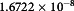	3.2509	0.4998
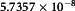	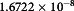	3.8720	0.5146
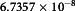	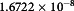	4.4931	0.5380
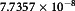	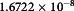	5.1143	0.5476
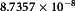	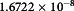	5.7354	0.5630
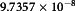	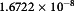	6.3565	0.5644
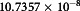	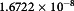	6.9777	0.5464
Baseline of 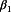 and 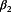			
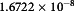	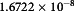	1.3480	0.2640
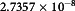	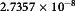	2.2054	0.5530
Only 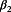 varies			
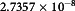	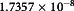	2.0204	0.4418
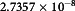	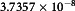	2.3904	0.6434
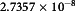	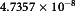	2.5754	0.6910
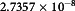	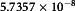	2.7604	0.7390
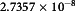	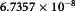	2.9454	0.7786
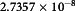	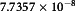	3.1304	0.7980
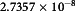	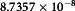	3.3154	0.8182
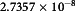	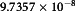	3.5004	0.8280
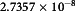	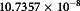	3.6854	0.8414

*Note*: Parameter values are given in Table 2.

## Discussion

Now we are in a world where interdisciplinary research is the most critical mechanism to live a healthy and standard life for all of humankind. Over the decades, mathematical prediction modeling used to be one of the essential tools to study and understand any epidemics’ behavior. It helps the policy-maker to make a crucial decision and get prepared for the coming days. This study’s aim was to develop a mathematical model with real-time data^24^ and predict the dynamics of the COVID-19 in the state of Texas.

We used the 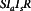 (modified SEIR) modeling framework to design our model. We estimated the model parameters using the data from Texas^24^ from March 6, 2020, to January 31, 2021. The average basic reproduction number, 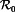, calculated as 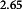 for the system Equation (2.3). A study by Liu et al. 2020^35^ calculates the median of 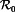 is 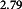 with a range from 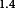 to 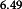.

Our model predicted that the net peak of daily new cases occurred at the first week of January, 2021, with new infections on the peak day (see Figure 2b). The epidemic will have another wave after April of 2021 infecting people in total during this period (Figure 3b).

According to the CDC, the incubation period of the COVID-19 is 2-14 d.^2^ Lauer et al.^36^ estimated the COVID-19 mean incubation period as 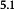 days. We considered the baseline incubation period of 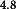 d in our model. The incubation period may be different for the nature of the population or the individual patient. A 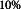 larger incubation period forces the peak epidemic size bigger and earlier with an 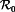 of 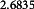 and a 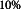 smaller incubation period delayed peak epidemic in a smaller size with 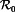 of 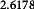 (see Figure 4).

Scientists worldwide are trying to find the proper control measures to slow the spread of the COVID-19 virus; social distancing, quarantine of the exposed individuals, isolation of infected persons, and wearing face masks are widely used strategies. The main target for containing the virus is to reduce the contact between susceptible and exposed/infected persons, which eventually will reduce the transmission rate from the susceptible class (*S*) to the infected classes (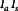). Texas closed schools, colleges, nonessential businesses during March 2020 and finally put the stay-at-home order at the beginning of April until the end of April 2020. This restriction delayed the earlier predicted peak on June 15 by Cooper et al.^5^ We concluded that it is more important to control the asymptomatically exposed individual’s contact with the population than the symptomatic infected person, as the symptomatic infected person already develops some symptoms. Hence, they know that they are contagious and restricted them from mixing with others. The asymptomatically exposed individual who does not have any symptoms may spread the virus even without knowing that they are contagious.

Our model suggests that the sensitivity of the transmission rate due to contact between susceptible and asymptomatically infected persons is 5 times greater than the transmission rate caused by the contact between susceptible to infected individuals. A 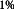 increase of the transmission rate from the susceptible population to the asymptomatically exposed population increases the 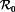 from 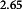 to 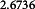, and 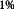 decrease the 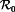 to 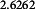 (see Figure 5 for epidemic size and the total number of infection). To get a similar change on 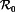, we had to increase/decrease the transmission rate from susceptible person to infected person, 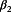 by 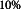 (see also Figure 6 for change in epidemic size and total infection). Due to the unavailability of the data, this article did not track the dynamics of the asymptomatic carrier. Tracking asymptomatic infection could provide additional insight into the dynamics of the disease.

A total of 250 of 254 counties reported SARS-CoV-2 infections, with nearly 2 million cases in all of Texas by January 31, 2021. Fifty-eight counties reported more than 1000 cases, 22 counties reported more than 5000 cases, and 11 counties reported more than 10,000 cases. In contrast, 89 counties reported less than 100 confirmed cases of coronavirus. It is noticeable that the top 6 infected counties (Harris, Dallas, Bexar, Tarrant, Travis) reported more than 50% of the total number of infections in Texas.^24^

County-wise daily case analysis for the top 15 (70% of Texas) infected counties (see Supplementary Tables A1, A2, A3, A4) shows that some counties (Harris, Dallas, Tarrant, Bexar, El Paso Travis) may pass their second epidemic peak. However, other counties (such as Montgomery, Williamson) still have an uptrend in daily new infections. Proper implementation of the health expert’s suggestions, such as using face coverings, maintaining social distance, and frequent hand washing, could play an important role in epidemic dynamics.

Additionally, the under-reporting of numerous daily deaths due cuased by coronavirus in different counties within the state again goes in line with the results of our 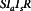 model. According to this simple yet effective exploratory prediction model, the early relaxation of lockdown (in April) within the state (overall in the United States) has potentially contributed to the exponential rise in COVID-19 cases. If the uptrend continues, the government and policy-makers have to rigorously prepare at a highly rapid pace for a large nationwide surge in patients presenting to intensive care units, also anticipating the daily rate at which front-line workers (both medical and law enforcement professionals) may succumb to death while serving the nation. The Texas governor may need to introduce draconian measures such as stay-at-home order and massive fines to limit population mobility and gatherings immediately.

The mathematical model has some limitations; our model has some as well. Texas has a different kind of epidemic scenario for different cities and counties; for instance, at a particular time, some cities and counties have stay-at-home order, other does not have the same restriction. The diversity of the population changes the contact behavior between population and changes the dynamics. Texas has has a mandatory mask policy since July 2, 2020.^37^ We did not consider those variations in our modeling approach. The next iteration of the modeling approach could implement the discussed idea to reveal additional insights into disease dynamics. Furthermore, SARS-CoV-2 is a novel coronavirus, and scientists all over the world discovered new information every day, which could lead to a new understanding of the disease dynamics.

## Conclusions

In conclusion, our 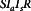 prediction model has successfully exhibited findings in line with the current practical situation regarding the ongoing and forthcoming COVID-19 epidemic transmission in Texas, USA. The state is already in an extremely critical situation, and the peak time near the door or passing. Learning from the experiences of other high-index countries with robust health systems, the government and policy-makers of Texas need to be extra vigilant and look for alternate, effective and aggressive measures for limiting the catastrophic impacts of the contagion and protecting the front-line workers. They risk their lives to provide essential services.

## Supplementary material

For supplementary material accompanying this paper visit http://dx.doi.org/10.1017/dmp.2021.151. click here to view supplementary material

## Appendix

All the supportive and supplementary results are presented in this section.

### Proof of Theorem 1

*Proof.* Equation (2.5) gives

This implies

(A.1)

Hence,  whenever

Hence, this inequality claims that  is bounded by .

Now, integrating the inequality in (A.1) and using the initial condition, we get

(A.2)

Now, when , we obtain  asymptotically.

Thus it has been established that, all components of the solution of Equation (2.3) are positive and bounded in the closed region.

### Proof of Theorem 2

*Proof.* The Jacobian matrix of the system Equation (2.3) at the DFE point is

Then the characteristic equation gives the eigenvalues as

and the other two eigenvalues are the roots of the following quadratic equation

where,

Here, two possible scenarios can happen:

1. If , then *B* obviously positive. Again  indicates that

Thus, . Then Routh-Hurwitz criterion for polynomials implies that DFE is stable.

2. If , then *B* obviously negative. Thus, . Again,  as . Since  is a continuous function of , hence by Bolzano’s theorem on continuous function we have  for some . Therefore, at least one eigenvalue of the Jacobian matrix is positive. Hence, DFE point is unstable equilibrium point.

This completes the proof.

### Proof of Theorem 3

*Proof.* The characteristic equation of the Jacobian matrix (3.9) at the EE is

where,  is an  identity matrix; which gives,

where,

Here, the first eigenvalue is negative, then the system will be locally asymptotically stable if other 3 eigenvalues are all negative or their real parts are negative. The other 3 eigenvalues will be negative or will have negative real parts if the Routh-Hurwitz criterion is satisfied.

From Routh-Hurwitz criterion, we can say that the EE point is stable if  and . Therefore the EE of Equation (3), which exists if  is locally asymptotically stable.
