# Supplementary material for: Mathematical Modeling and COVID-19 Forecast in Texas, USA: A Prediction Model Analysis and the Probability of Disease Outbreak
Source: Disaster Med Public Health Prep. 2021 May 19:1–12. doi: 10.1017/dmp.2021.151 (PMC8314068; doi:10.1017/dmp.2021.151)
Supplement: Supplementary file 1 [file dmpsup.zip › S1935789321001518sup001.pdf]

County	Harris	Dallas	Tarrant	Bexar	El Paso	Travis	Collin	Fort Bend	Lubbock	Hidalgo	Denton	Webb	Cameron	Montgome	Williamson
1-May	195	179	97	48	37	63	14	34	19	10	17	5	11	20	8
2-May	195	187	142	103	37	29	42	27	8	5	21	5	9	27	5
3-May	157	181	115	108	25	31	18	54	6	1	13	17	11	5	7
4-May	130	234	81	28	12	42	5	50	4	1	4	6	0	0	10
5-May	129	237	40	39	31	60	15	4	7	12	3	5	11	6	5
6-May	161	253	62	25	51	60	24	58	7	0	26	4	11	22	5
7-May	116	246	127	84	39	70	11	42	8	6	14	10	15	10	8
8-May	133	251	143	44	71	56	18	45	7	9	26	4	14	17	11
9-May	217	249	171	30	86	69	30	25	1	9	30	10	11	10	9
10-May	209	250	83	66	46	24	14	13	6	10	12	10	18	0	7
11-May	75	251	485	0	18	32	17	15	3	2	8	1	0	0	9
12-May	298	253	50	19	8	44	21	19	6	5	24	2	15	25	3
13-May	240	236	65	22	65	64	24	0	7	9	22	7	17	18	17
14-May	205	243	266	34	43	53	15	48	2	6	21	1	25	16	27
15-May	196	235	135	65	151	57	15	19	7	8	27	6	13	15	18
16-May	233	199	55	79	48	36	15	39	2	8	24	18	0	25	9
17-May	76	214	84	0	71	44	8	48	3	0	11	8	37	0	12
18-May	339	205	97	0	46	34	12	1	0	15	18	0	0	0	18
19-May	170	224	112	72	80	78	19	0	5	8	19	2	16	19	5
20-May	224	225	84	86	78	58	26	22	6	9	28	13	14	31	15
21-May	236	0	68	44	116	49	17	40	9	10	45	6	15	13	12
22-May	188	369	92	49	114	68	28	31	11	5	27	2	0	20	7
23-May	243	204	96	21	105	49	14	9	6	10	17	2	22	7	8
24-May	244	172	52	26	75	27	4	15	6	0	7	9	12	0	13
25-May	151	178	26	24	36	59	15	9	2	15	12	5	0	0	12
26-May	74	171	0	7	17	86	6	47	3	7	9	0	0	0	12
27-May	286	190	62	31	68	51	32	0	7	12	22	5	13	25	19
28-May	261	197	151	45	108	73	28	34	3	21	30	3	20	13	4
29-May	228	202	104	58	54	67	19	17	3	0	33	11	12	14	8
30-May	239	200	85	53	81	62	42	49	8	11	11	5	11	14	9
31-May	211	219	84	189	60	46	19	29	3	12	28	6	10	0	7
1-Jun	56	228	50	5	30	40	15	20	1	15	16	2	0	0	9
2-Jun	388	228	21	9	39	88	0	0	12	9	25	3	12	12	9
3-Jun	363	257	89	43	39	73	34	35	2	26	18	1	23	44	6
4-Jun	241	239	138	71	197	68	42	35	1	24	19	13	21	23	13
5-Jun	335	285	102	65	144	61	42	81	13	45	19	9	21	20	11
6-Jun	337	298	122	125	85	54	17	85	14	0	13	20	22	11	12
7-Jun	483	289	88	147	78	46	0	0	7	22	19	0	29	0	12
8-Jun	163	263	77	21	0	35	0	0	6	0	14	0	0	0	7
9-Jun	332	254	219	22	136	118	81	21	9	58	24	14	27	26	7
10-Jun	320	298	64	180	70	161	18	87	3	45	35	26	32	32	47
11-Jun	314	300	166	135	68	133	7	74	16	38	40	18	39	40	18
12-Jun	312	312	225	192	45	129	7	21	15	70	33	9	41	35	24
13-Jun	324	328	296	172	99	76	78	38	23	55	55	14	50	61	33
14-Jun	380	345	133	230	40	147	9	27	34	0	32	0	57	0	42
15-Jun	210	302	81	151	77	84	20	0	10	83	17	0	0	0	42
16-Jun	504	305	164	44	37	119	120	17	37	62	36	43	40	42	56
17-Jun	425	306	144	439	51	107	79	94	61	143	40	38	58	59	61
18-Jun	450	413	180	266	89	220	101	171	76	92	81	47	65	69	47
19-Jun	395	392	277	408	90	0	59	59	105	100	87	51	70	56	46
20-Jun	1187	394	232	412	162	295	38	156	72	109	74	20	83	71	45
21-Jun	1135	395	180	382	148	418	125	69	80	0	59	0	92	0	78
22-Jun	179	408	444	538	65	506	34	118	32	133	50	55	0	0	100
23-Jun	1994	454	171	274	124	129	27	63	87	193	56	19	101	92	74
24-Jun	1374	445	260	311	132	257	109	44	140	248	83	55	111	90	87
25-Jun	1365	391	460	347	175	318	90	91	118	373	115	42	123	94	116
26-Jun	1231	403	517	638	233	183	37	130	130	210	77	56	91	58	88
27-Jun	1238	496	343	405	113	0	46	248	121	179	87	111	113	63	68
28-Jun	908	561	377	795	284	728	139	71	68	0	49	50	98	0	80
29-Jun	113	570	393	495	131	636	92	0	57	402	40	195	0	0	169
30-Jun	1453	572	263	650	183	508	119	6	54	248	70	46	113	76	81
1-Jul	693	601	605	1268	196	558	115	60	178	440	105	35	103	105	130
2-Jul	1437	544	606	439	265	597	117	86	184	270	106	66	111	124	109
3-Jul	1249	708	473	374	276	571	85	131	163	268	81	99	101	96	49
4-Jul	1208	1085	0	1334	288	314	122	121	0	278	152	70	81	0	81
5-Jul	597	1103	0	341	0	122	88	0	0	0	95	58	87	0	0
6-Jul	684	1062	585	198	508	548	112	0	0	547	59	37	0	0	0
7-Jul	1179	1214	820	608	181	247	186	166	298	0	65	92	101	197	682
8-Jul	1535	1077	757	778	393	482	118	60	133	102	56	131	117	104	0
9-Jul	701	1029	595	845	350	753	123	67	180	335	123	72	123	130	291
10-Jul	907	1201	520	954	361	703	160	111	145	278	115	242	126	92	175
11-Jul	1081	1164	634	923	353	440	200	93	148	1274	113	142	250	131	37
12-Jul	1939	1101	423	535	411	318	151	141	144	393	127	175	358	0	91
