# Supplementary material for: Mathematical Modeling and COVID-19 Forecast in Texas, USA: A Prediction Model Analysis and the Probability of Disease Outbreak
Source: Disaster Med Public Health Prep. 2021 May 19:1–12. doi: 10.1017/dmp.2021.151 (PMC8314068; doi:10.1017/dmp.2021.151)
Supplement: Supplementary file 1 [file dmpsup.zip › S1935789321001518sup002.pdf]

County	Harris	Dallas	Tarrant	Bexar	El Paso	Travis	Collin	Fort Bend	Lubbock	Hidalgo	Denton	Webb	Cameron	Montgome	Williamson
6-Dec	1027	1411	1020	444	217	359	692	983	371	0	327	537	0	0	0
7-Dec	2192	1787	1440	900	453	284	313	0	277	0	0	228	0	0	0
8-Dec	515	1606	1613	1030	500	272	235	311	164	0	495	91	261	408	521
9-Dec	745	860	712	985	381	355	428	473	192	386	458	0	101	140	294
10-Dec	941	1090	1240	468	275	256	493	1074	345	676	623	830	84	172	176
11-Dec	1266	1717	949	927	348	427	506	221	261	0	612	123	157	120	225
12-Dec	1896	1353	1277	899	608	382	583	133	293	660	724	45	113	246	234
13-Dec	1000	669	1109	742	219	235	426	132	284	0	368	165	0	0	0
14-Dec	1416	2551	1286	568	405	305	586	0	350	453	0	109	0	0	0
15-Dec	1264	1519	1183	475	188	415	654	178	322	0	682	112	294	457	497
16-Dec	1489	1549	1030	1164	380	613	655	81	595	447	717	160	157	296	363
17-Dec	2265	1942	1552	1093	350	367	644	737	258	152	540	372	184	281	371
18-Dec	991	976	1374	1234	247	545	656	223	487	0	628	220	187	138	293
19-Dec	1140	1938	1725	638	339	415	384	417	268	185	506	192	139	196	234
20-Dec	1266	0	1601	504	251	274	417	100	349	0	295	0	0	0	0
21-Dec	2097	2164	644	472	357	422	717	0	318	0	0	505	0	0	0
22-Dec	2481	1655	1332	1140	214	537	959	128	290	254	456	0	361	0	784
23-Dec	988	2088	1288	1401	291	672	1113	1317	352	130	366	495	0	1045	564
24-Dec	1231	1835	1404	1328	319	375	797	352	399	148	487	0	127	394	0
25-Dec	838	0	0	0	264	0	0	0	204	169	0	599	135	0	0
26-Dec	263	0	0	0	326	0	785	0	0	0	0	0	0	0	0
27-Dec	73	1089	0	0	114	1132	476	0	476	0	408	0	0	0	0
28-Dec	5340	0	1865	3928	0	216	359	0	105	0	0	361	0	0	0
29-Dec	1623	6622	5441	956	280	527	897	404	171	178	370	268	446	0	951
30-Dec	1278	882	1309	1804	267	697	479	1212	398	152	631	0	91	1708	549
31-Dec	1754	1965	1354	1035	496	546	493	346	275	342	453	678	113	192	343
1-Jan	1036	1418	2536	0	338	401	803	166	256	274	401	146	0	0	0
2-Jan	1677	0	0	0	393	0	563	0	0	0	0	22	147	0	0
3-Jan	3402	0	2865	3137	343	963	940	0	490	0	492	0	0	0	0
4-Jan	2492	6009	3507	1907	215	398	409	0	65	0	0	0	0	0	0
5-Jan	1777	1420	2234	1018	531	752	997	202	262	133	359	0	620	1549	1477
6-Jan	1927	2562	1574	1613	511	564	771	720	315	385	418	404	173	371	548
7-Jan	1235	1818	2777	1636	376	663	636	1362	287	386	478	293	303	350	400
8-Jan	2589	2207	1926	775	593	879	434	392	176	498	512	0	201	243	324
9-Jan	2164	2106	2448	750	808	592	761	340	366	659	433	1035	223	177	254
10-Jan	3536	2293	2513	1167	301	464	628	0	143	0	296	0	0	0	0
11-Jan	2579	1987	2482	2718	590	0	836	0	173	0	0	880	0	0	0
12-Jan	3619	1813	1906	1355	298	955	692	376	136	351	391	0	155	990	1035
13-Jan	1157	2979	1498	2022	564	0	726	964	210	247	462	0	126	587	459
14-Jan	1341	2589	1547	890	633	1461	716	347	140	505	503	3057	703	187	291
15-Jan	5614	1796	1943	1600	422	1108	288	859	170	501	381	0	277	326	471
16-Jan	3125	2153	2020	2600	543	690	638	789	136	397	345	0	245	285	595
17-Jan	2842	2432	2745	2597	359	295	587	0	105	0	280	1664	0	0	0
18-Jan	3276	1311	1343	1549	496	403	555	0	182	0	0	177	0	0	0
19-Jan	1417	0	2686	0	353	686	794	405	110	0	0	191	0	0	0
20-Jan	1202	1351	1841	3241	453	834	353	1313	117	447	739	878	359	1171	1454
21-Jan	2263	3197	1770	607	556	734	479	765	159	492	587	1022	247	201	397
22-Jan	2912	1412	1527	2343	547	715	781	112	118	470	548	564	213	292	568
23-Jan	803	1843	1480	2054	0	540	767	265	110	379	471	607	231	0	295
24-Jan	1884	1525	1854	1561	981	367	231	0	59	0	159	0	0	0	0
25-Jan	931	1071	1442	200	0	305	775	0	78	0	0	0	0	0	0
26-Jan	3186	1476	1061	1809	936	544	166	406	88	386	370	691	511	2137	763
27-Jan	2936	1497	1072	573	628	702	673	888	123	284	514	0	223	102	296
28-Jan	1874	1397	982	1195	530	650	616	351	120	400	434	471	108	416	431
29-Jan	2130	1379	2261	1604	368	707	526	448	133	404	476	0	101	226	369
30-Jan	2314	1494	1028	1444	464	454	331	463	97	451	395	1069	150	223	433
31-Jan	2756	1170	1560	1980	480	270	325	0	60	0	183	62	0	0	0
