# Supplementary material for: Mathematical Modeling and COVID-19 Forecast in Texas, USA: A Prediction Model Analysis and the Probability of Disease Outbreak
Source: Disaster Med Public Health Prep. 2021 May 19:1–12. doi: 10.1017/dmp.2021.151 (PMC8314068; doi:10.1017/dmp.2021.151)
Supplement: Supplementary file 1 [file dmpsup.zip › S1935789321001518sup003.pdf]

County	Harris	Dallas	Tarrant	Bexar	El Paso	Travis	Collin	Fort Bend	Lubbock	Hidalgo	Denton	Webb	Cameron	Montgome	Williamson
13-Jul	1429	1174	404	511	206	166	74	41	80	313	112	158	0	0	65
14-Jul	2001	1114	322	565	237	657	152	216	75	0	113	121	321	105	232
15-Jul	1658	1000	531	729	345	553	115	196	0	157	154	210	415	359	111
16-Jul	1343	1055	857	454	340	572	134	73	240	396	151	162	315	853	174
17-Jul	1599	1027	562	5268	205	413	145	87	146	651	138	201	335	177	93
18-Jul	1586	1195	474	459	289	232	75	318	100	1248	160	103	325	220	70
19-Jul	1251	1031	288	867	441	239	137	290	136	451	122	152	326	0	196
20-Jul	963	1044	422	1985	196	192	83	0	37	1320	81	121	0	0	106
21-Jul	1326	1026	385	370	272	145	82	16	74	524	130	181	322	346	96
22-Jul	1385	734	663	1064	256	603	158	135	100	339	218	145	307	102	93
23-Jul	1444	413	871	851	204	302	134	109	113	650	228	196	334	125	90
24-Jul	1492	648	431	716	249	243	-177	113	111	813	179	227	308	150	74
25-Jul	1203	659	595	334	221	238	111	108	82	564	192	173	684	153	58
26-Jul	1494	1267	584	493	269	224	80	70	52	0	128	170	-19	0	43
27-Jul	1236	800	353	210	0	79	27	0	86	0	78	39	0	0	58
28-Jul	846	426	240	41	312	240	149	149	50	606	87	55	374	183	62
29-Jul	1465	789	576	1234	255	232	209	93	104	329	123	260	576	72	67
30-Jul	1466	704	836	682	132	235	30	80	74	287	162	280	647	138	118
31-Jul	1724	537	672	1029	337	278	76	108	105	271	188	202	731	87	65
1-Aug	2114	707	587	420	134	280	100	122	79	360	122	253	816	119	99
2-Aug	1920	614	461	250	302	222	47	109	42	0	91	229	1106	0	0
3-Aug	1758	518	183	471	202	247	3	0	39	347	44	51	1439	0	0
4-Aug	1463	382	303	36	228	335	18	96	59	398	94	218	1265	95	117
5-Aug	1438	641	805	82	285	273	317	580	99	498	95	292	337	106	98
6-Aug	1371	508	65	235	169	202	119	692	60	450	130	198	446	62	19
7-Aug	1005	230	423	214	312	232	217	319	148	404	117	298	301	51	109
8-Aug	1264	422	272	227	132	224	194	273	59	431	145	123	425	67	187
9-Aug	1417	540	765	123	268	122	171	382	0	0	67	285	0	0	0
10-Aug	1157	843	490	168	88	122	215	0	41	207	58	63	300	0	0
11-Aug	806	581	292	98	540	229	179	488	163	407	101	374	316	77	648
12-Aug	942	298	197	134	274	296	219	314	73	283	122	228	163	122	118
13-Aug	989	234	404	236	168	172	261	152	70	336	92	324	247	44	64
14-Aug	931	641	333	150	254	297	132	336	64	508	118	312	302	44	73
15-Aug	1149	885	471	97	447	152	1167	546	66	531	137	177	198	93	182
16-Aug	1124	754	1436	123	271	138	395	859	33	0	86	132	0	0	0
17-Aug	555	166	687	43	136	136	-1	0	14	207	0	117	189	0	0
18-Aug	691	1850	242	45	196	265	163	395	0	0	127	94	165	203	152
19-Aug	928	237	451	86	232	350	210	411	114	813	157	211	168	167	99
20-Aug	804	355	330	0	143	303	33	235	50	290	106	100	221	206	43
21-Aug	955	102	190	267	120	291	39	83	44	397	151	90	215	46	22
22-Aug	1027	255	197	0	139	206	0	106	68	480	109	40	227	38	41
23-Aug	1087	224	215	419	80	81	0	240	34	0	145	7	224	0	0
24-Aug	761	239	212	84	158	96	0	0	33	501	0	36	0	0	0
25-Aug	784	98	90	73	68	154	23	606	33	675	140	0	637	36	60
26-Aug	881	217	359	96	131	127	96	5	57	782	171	215	0	52	0
27-Aug	906	154	168	95	172	100	215	116	60	655	138	72	755	130	73
28-Aug	593	160	193	153	147	122	27	0	138	471	201	17	122	0	47
29-Aug	1418	258	202	237	145	68	77	84	69	175	130	48	114	53	26
30-Aug	1561	369	202	173	133	9	57	88	102	0	119	251	0	0	0
31-Aug	1108	119	168	105	110	118	43	0	53	156	0	81	0	0	0
1-Sep	838	225	118	18	79	82	65	24	88	130	131	31	195	143	56
2-Sep	895	455	154	199	72	7	65	26	269	131	114	249	163	54	20
3-Sep	595	323	171	191	75	99	79	53	273	239	101	295	131	77	15
4-Sep	734	249	160	140	0	104	108	45	304	270	82	253	118	114	22
5-Sep	1015	165	154	75	164	128	118	19	275	191	92	186	103	118	18
6-Sep	928	203	249	100	89	77	57	44	261	0	74	197	0	0	0
7-Sep	763	244	0	207	154	38	53	0	45	222	0	69	0	0	0
8-Sep	0	261	0	0	94	69	98	0	71	141	51	37	0	0	0
9-Sep	514	139	221	161	72	86	44	14	92	161	127	13	239	45	68
10-Sep	723	266	350	98	76	82	58	91	202	189	81	227	31	33	15
11-Sep	742	152	157	51	0	218	70	82	125	0	69	93	110	61	18
12-Sep	948	195	168	103	224	101	182	21	98	253	61	68	40	204	18
13-Sep	388	160	155	58	62	53	15	71	116	0	37	48	0	0	0
14-Sep	1023	139	294	101	110	123	238	0	33	111	0	77	0	0	0
15-Sep	1080	340	256	78	95	75	130	48	179	73	116	19	169	116	84
16-Sep	357	122	385	95	0	185	87	28	0	202	95	57	70	57	25
17-Sep	268	298	139	123	252	142	102	126	216	325	92	77	54	68	44
18-Sep	552	406	230	103	101	109	67	53	75	329	65	174	63	59	35
19-Sep	477	329	239	121	150	163	55	56	118	202	59	69	66	14	54
20-Sep	612	155	309	146	147	63	123	55	75	0	60	75	0	0	0
21-Sep	225	465	203	103	176	3	42	0	143	0	0	43	0	108	0
22-Sep	507	314	302	71	169	155	49	25	60	166	106	30	94	0	73
23-Sep	142	170	274	165	0	47	125	94	166	0	95	168	0	-29	18
