# Supplementary material for: Mathematical Modeling and COVID-19 Forecast in Texas, USA: A Prediction Model Analysis and the Probability of Disease Outbreak
Source: Disaster Med Public Health Prep. 2021 May 19:1–12. doi: 10.1017/dmp.2021.151 (PMC8314068; doi:10.1017/dmp.2021.151)
Supplement: Supplementary file 1 [file dmpsup.zip › S1935789321001518sup004.pdf]

County	Harris	Dallas	Tarrant	Bexar	El Paso	Travis	Collin	Fort Bend	Lubbock	Hidalgo	Denton	Webb	Cameron	Montgome	Williamson
24-Sep	106	326	322	105	386	102	83	141	174	419	68	105	87	14	39
25-Sep	204	140	225	154	181	159	161	53	156	209	91	28	47	34	0
26-Sep	252	295	246	185	216	169	38	65	230	191	89	149	65	0	50
27-Sep	113	202	240	69	180	57	24	40	83	0	35	35	0	49	0
28-Sep	172	197	180	38	121	122	228	0	98	0	0	11	0	0	0
29-Sep	133	186	229	51	265	91	54	88	342	115	73	78	83	64	59
30-Sep	300	758	447	390	276	78	97	92	188	158	90	109	44	70	18
1-Oct	141	247	323	125	220	93	187	85	187	149	94	18	51	72	21
2-Oct	81	0	257	89	333	62	45	22	103	214	77	98	41	115	9
3-Oct	266	508	304	82	207	71	192	78	156	208	64	90	29	72	34
4-Oct	380	249	382	114	363	103	20	31	182	0	42	0	0	0	0
5-Oct	214	453	482	106	224	49	41	0	60	0	0	55	0	0	0
6-Oct	171	450	208	60	237	58	126	17	126	214	83	11	83	105	96
7-Oct	192	246	224	117	402	95	78	22	173	193	59	131	55	200	59
8-Oct	191	339	438	160	537	45	109	61	131	205	79	14	43	161	21
9-Oct	129	198	359	106	425	78	83	9	213	130	74	19	20	149	12
10-Oct	248	415	278	125	559	90	141	85	232	204	108	44	165	77	23
11-Oct	299	455	335	0	546	34	57	83	241	0	36	0	0	0	0
12-Oct	420	395	683	108	0	66	74	0	250	0	0	0	0	0	0
13-Oct	223	446	289	42	885	133	101	32	159	143	96	100	180	223	86
14-Oct	167	597	454	110	503	0	104	54	212	119	87	9	67	115	40
15-Oct	646	443	436	66	0	203	100	53	298	174	145	15	53	61	31
16-Oct	211	529	522	151	1555	87	163	19	246	246	114	181	50	0	19
17-Oct	318	462	494	142	558	109	108	24	253	145	157	0	0	205	44
18-Oct	229	593	501	133	698	111	75	64	227	0	84	0	47	0	0
19-Oct	219	516	396	90	555	48	0	0	225	0	0	0	0	0	0
20-Oct	300	329	307	86	666	97	111	31	282	0	114	164	109	82	0
21-Oct	223	472	395	74	670	106	74	81	222	164	112	76	53	0	0
22-Oct	637	522	513	143	1161	69	126	113	287	0	131	40	35	0	126
23-Oct	280	350	660	140	969	99	195	11	185	456	98	41	31	400	26
24-Oct	357	531	678	176	1216	84	168	30	309	171	155	21	39	110	46
25-Oct	732	648	660	150	517	82	79	0	230	0	103	0	0	0	0
26-Oct	276	703	595	122	1443	58	78	0	337	0	0	95	0	0	0
27-Oct	385	391	422	122	1390	102	115	94	225	130	167	39	109	130	83
28-Oct	170	418	401	98	854	122	132	70	312	181	120	30	65	94	61
29-Oct	741	479	533	226	1128	76	232	29	697	204	125	124	30	89	30
30-Oct	303	565	483	155	1347	92	115	49	273	177	97	72	41	112	20
31-Oct	518	670	611	132	1643	81	273	42	182	123	115	42	59	50	59
1-Nov	629	622	496	219	1146	119	112	59	251	0	94	0	0	0	0
2-Nov	365	700	657	159	1923	77	96	0	333	0	0	64	0	0	0
3-Nov	409	476	446	122	1867	83	61	28	577	160	161	25	100	83	76
4-Nov	447	598	571	210	1537	148	284	44	460	202	133	37	43	56	44
5-Nov	496	687	577	133	1920	154	291	55	531	130	143	117	55	38	47
6-Nov	635	601	795	127	1300	143	221	163	427	216	151	112	83	30	60
7-Nov	615	867	819	131	1020	180	109	65	438	193	171	41	37	0	33
8-Nov	458	654	782	131	1919	88	355	45	139	0	40	0	0	0	0
9-Nov	650	807	351	46	899	152	99	0	311	0	0	352	0	0	0
10-Nov	493	1095	1415	334	1292	214	142	0	514	198	190	66	149	0	163
11-Nov	661	1267	850	223	863	177	270	114	325	147	179	331	78	0	0
12-Nov	325	926	684	0	976	210	146	0	440	183	204	107	0	0	68
13-Nov	843	411	885	425	1488	191	229	161	424	0	141	27	70	0	96
14-Nov	711	499	1180	263	1512	244	167	210	509	199	184	273	97	0	70
15-Nov	624	1454	700	260	981	300	234	0	192	0	211	199	0	0	0
16-Nov	1027	1281	1318	188	1550	108	0	141	772	0	0	111	0	0	0
17-Nov	262	1695	808	146	994	157	23	24	335	167	271	175	167	0	174
18-Nov	450	457	577	87	927	251	289	88	548	426	190	365	75	0	68
19-Nov	1458	786	1946	203	672	306	353	99	412	681	206	294	74	729	100
20-Nov	1275	1804	1598	356	1062	290	26	123	555	679	284	0	88	0	126
21-Nov	1014	1646	1171	796	1074	368	322	128	474	719	304	260	111	484	249
22-Nov	1371	1741	1248	355	843	191	690	129	241	0	241	336	0	0	0
23-Nov	727	1811	1443	536	328	261	403	0	498	0	0	116	0	0	0
24-Nov	873	521	986	608	1257	318	540	228	505	0	332	65	194	0	376
25-Nov	1281	1516	1281	830	587	366	241	138	676	1384	305	318	0	0	337
26-Nov	1766	1185	1143	820	542	118	311	260	538	763	339	146	154	0	0
27-Nov	558	0	0	0	678	0	271	0	319	0	0	150	0	0	0
28-Nov	105	0	0	0	590	428	413	0	588	612	250	110	0	0	0
29-Nov	60	739	0	1496	346	232	626	462	359	0	147	93	0	0	0
30-Nov	2614	3083	1042	1475	461	147	408	0	378	0	0	0	0	0	0
1-Dec	810	684	3172	298	319	332	390	127	190	176	297	0	296	1994	1029
2-Dec	583	1096	1216	265	551	380	561	172	619	526	331	577	139	269	214
3-Dec	903	1407	1541	1145	545	280	295	760	226	607	358	0	52	190	159
4-Dec	1734	1637	1611	492	570	230	257	602	593	726	381	451	124	0	160
5-Dec	557	649	1270	1176	416	299	442	1285	210	508	454	0	109	0	178
